# Improved YOLO Based Detection Algorithm for Floating Debris in Waterway

**DOI:** 10.3390/e23091111

**Published:** 2021-08-27

**Authors:** Feng Lin, Tian Hou, Qiannan Jin, Aiju You

**Affiliations:** 1 College of Electrical Engineering, Zhejiang University, Hangzhou 310027, China; houtian@zju.edu.cn; 2 Zhejiang Institute of Hydraulics and Estuary, Hangzhou 310020, China; jqn.amy@126.com (Q.J.); yajyaj1997@126.com (A.Y.)

**Keywords:** deep learning, YOLOv5, floating debris detection, detection algorithm

## Abstract

Various floating debris in the waterway can be used as one kind of visual index to measure the water quality. The traditional image processing method is difficult to meet the requirements of real-time monitoring of floating debris in the waterway due to the complexity of the environment, such as reflection of sunlight, obstacles of water plants, a large difference between the near and far target scale, and so on. To address these issues, an improved YOLOv5s (FMA-YOLOv5s) algorithm by adding a feature map attention (FMA) layer at the end of the backbone is proposed. The mosaic data augmentation is applied to enhance the detection effect of small targets in training. A data expansion method is introduced to expand the training dataset from 1920 to 4800, which fuses the labeled target objects extracted from the original training dataset and the background images of the clean river surface in the actual scene. The comparisons of accuracy and rapidity of six models of this algorithm are completed. The experiment proves that it meets the standards of real-time object detection.

## 1. Introduction

With the development of the city and the increase in population, people’s requirements for the environment are constantly improving. As the largest water area on the earth’s surface, the ocean has been shown to have great economic and ecological value in reducing the number of floating debris [1,2], and researchers put forward relevant detection algorithms according to the characteristics of floating debris in the ocean [3,4,5,6]. The above researches are to classify the floating objects by using satellite images, which require high image acquisition equipment. Additionally, the detected objects are large aggregated floating objects, which is not suitable for the detection of a single relatively small object. However, the environment of the river is very different from that of the ocean. The floating debris in waterways has a great impact on the beauty of the river, the water quality of the river and the evaluation of the environment; furthermore, it has an important influence on the floating debris in the ocean too [7]. The main sources of garbage in the water area are domestic garbage, crop straw, tree branches and leaves, and surface vegetation, and so on. It is characterized by regular changes in seasons and climate. Generally, the flood season is the peak period, and the rainy season is more than the dry season. Among the non-degradable floating debris, various kinds of bottle floating objects account for the majority [8]. The quantity and species of floating debris in the waterway can reflect the water quality to a certain extent and can be used as an index to measure the water quality. The floating objects in rivers are often found by watching a video or directly at the scene; it takes a lot of labor and time. With the development of computer vision technology, it is possible to automatically identify and monitor the floating debris based on computer vision.

Traditional image processing methods include the mean-shift search model and Kalman filter prediction model to complete the tracking of moving targets [9]. The image is filtered and analyzed, and then the shape, color, texture and other features of the target are extracted. Finally, the collected features are classified by support vector machine (SVM) [10]. The K-nearest neighbor algorithm is used to establish the background model, and then the image and the background model are differentiated to detect the foreground target [11]. Kataoka et al. [12] used a traditional image processing approach to study the transport of riverine macro-debris quantitatively. Although this kind of algorithm does not need a large number of image data, its robustness is poor, and it is easy to be affected by the factors such as reflection and river grass occlusion; identifying the floating debris in the complex water area is not suitable.

With the improvement of data volume and the performance of computing equipment, the deep learning algorithm based on a convolutional neural network shows its advantages. The deep learning method proved to be superior to the existing machine learning technology in many fields, among which target detection is one of the most prominent examples [13,14]. Deep learning algorithms can be divided into two categories: one is the two-stage region-convolutional neural network(R-CNN) [15,16,17,18] series algorithm, the other is the one-stage single shot multi-box detector (SSD) [19] and you only look once (YOLO) [20,21,22,23] series algorithm.

The latest researches show that YOLO series algorithms can be applied to different detection scenarios. Zheng et al. [24] proposed a convolution neural network based on YOLOv3 to detect bearing-cover defects. Teng et al. [25] used a well-known feature extractor model and the YOLOv2 network to detect a concrete crack. Li et al. [26] proposed an enhanced YOLOv3 tiny network for real-time ship detection. Yolo is also widely used in radar images and remote sensing images. For instance, both Chen et al. [27] and Zhou et al. [28] used radar images as training datasets, and their detectors achieved lighter architecture and real-time detection speed. Gao et al. [29] developed a novel CNN model named YOLO-S-*CIOU*, which is based on YOLOv3 for specific building detection. In [30], the YOLO detector was trained on a thermal image dataset for person detection. In [31], the authors proposed a ratio-and-scale aware YOLO (RSA-YOLO) to solve the ratio problems. In the field of deep learning, annotation is also a very important problem. In supervised learning, all data in the training dataset have corresponding annotation information. However, there will be some wrong annotation or missing annotation. In order to make full use of the data in the training dataset, semi-supervised learning is also a common learning method. In image classification and target detection, semi-supervised learning can be used in the case of insufficient annotation of the training dataset [32,33].

**Figure 1:**
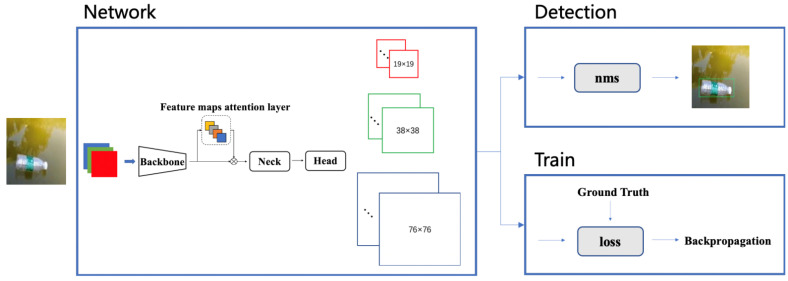
Overall framework of FMA-YOLOv5s algorithm.

In this paper, an improved YOLOv5s (FMA-YOLOv5s) algorithm by adding a feature map attention (FMA) layer at the end of the backbone is proposed to enhance the ability of network feature extraction. The FMA layer does not change the size of the input feature map so that it can be easily added to any network structure with flexibility and versatility. The network structure is used to detect the floating debris in the river. The main work of this article is as follows:

1. The network structure of YOLOv5s was redesigned, and a feature map attention (FMA) layer was added between the backbone and neck, as shown in Figure 1. In this layer, the input feature map was weighted to improve the feature extraction ability of the lightweight object detection YOLOv5s backbone; nevertheless, the number of channels of the feature map was not increased, and the calculation of the subsequent neck and head parts remained unchanged. The number of network parameters is small, and the detection speed and accuracy are high. This new network is called FMA-YOLOv5s in this paper;
2. The mosaic data augmentation is introduced in training, which can raise the detection ability of small targets on the water. The four images are randomly stitched together by mosaic data augmentation, and then they are sent into the model. On the one hand, it increases the batch size indirectly, which makes each iteration receives more data and speed up the training. On the other hand, it also reduces the size of the target, which makes the training dataset contain more small targets, and therefore raises the detection efficiency of small targets in the river;
3. A novel method to expand the training dataset is presented. This approach can quickly and effectively expand the number of images in the training dataset, especially for the scene where the target object is difficult to collect. This method separates the target objects and the background: the target objects in the training dataset are cropped; the clean river surface images are collected as the background images. Subsequently, those target objects and background images are merged by Poisson Blending to obtain a new dataset. Experiments show that the performance of the method in the test dataset was significantly improved;
4. Compared with the other six object detection networks, the experiment shows that the FMA-YOLOv5s network structure has good detection accuracy while ensuring detection speed.

## 2. Implementation of FMA-YOLOv5 Object Detection Algorithm

An algorithm based on FMA-YOLOv5 was designed to implement the real-time floating debris detection in this paper. The algorithm is composed of two processes: network training and detection, as shown in Figure 1. The weight parameters of the model are continuously updated by the loss of backpropagation in the training process. The weight value of the model does not change during detection. The prediction value is filtered according to the threshold value, and the final detection result is selected and marked on the image.

### 2.1. YOLOv5 Network Structure

**Figure 2:**
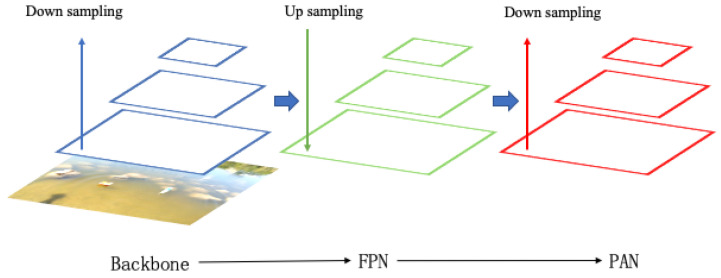
YOLOv5 network structure.

The training and detection network of this algorithm adopts the YOLOv5 network structure, as shown in Figure 2. The backbone part of the network is Cross Stage Partial Network (CSPNet) [34]. CSPNet alleviates the problem that requires a lot of inference calculations. CSPNet divides the feature map of the base layer into two parts and then extracts the image features by merging the cross-stage hierarchical structure. The advantage of this method is that it reduces the repeated gradient information, decreases the amount of calculation, improves the calculation speed of the equipment and does not affect the accuracy of the model. In order to make full use of the feature information extracted from different layers, YOLOv5 also adopts the network structure of feature pyramid networks (FPN) [35]. The feature maps of different levels obtained from the downsampling of the input image are then processed in upsampling from top to bottom, and the new feature map is obtained by splicing with the original feature map on the left. This structure not only enriches the scale of the output feature map but also achieves a better effect by combining shallow information with deep information. After the FPN feature combination, the path aggregation network(PAN) [36] structure is added on this basis. After convolution downsampling, the combined bottom feature map is spliced with the same scale feature map in the left FPN structure, and finally, three output feature maps with different scales are obtained. The purpose of this combination is to convey strong location features from the bottom up and increase the accuracy of the model’s feature extraction.

The three scale feature maps of the model are 19 × 19, 38 × 38 and 76 × 76, respectively. Among them, the feature map with the size of 19 × 19 has a larger downsampling ratio, which is suitable for the target with a larger scale, while the feature map with the size of 76 × 76 has a smaller downsampling ratio, which is suitable for the target with a smaller scale. Each pixel of the feature map predicts the correction value of three sets of prior frames, one confidence and eight targets. The total number of channels is 39 = 3 × (4 + 1 + 8).

### 2.2. Feature Map Attention Layer Structure

**Figure 3:**
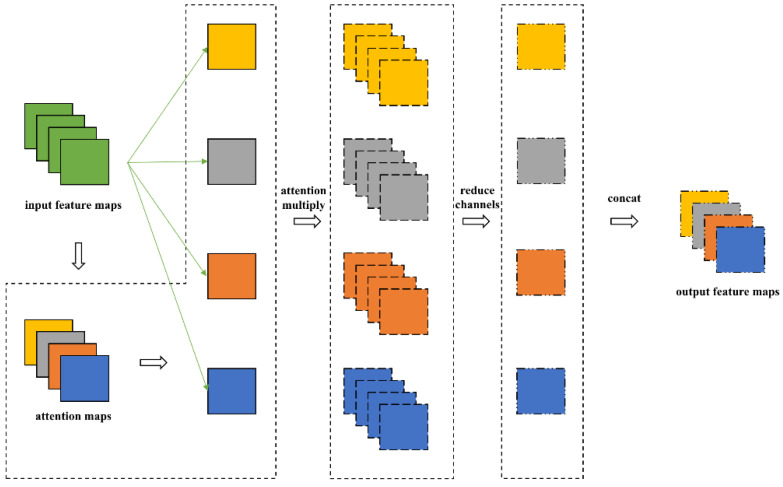
Feature map attention (FMA) layer.

The YOLOv5 algorithm is selected as the network model of floating debris detection. Compared with YOLOv2, YOLOv3 and YOLOv4, YOLOv5 has a more flexible network structure, which can adjust the network structure of different tasks more conveniently. YOLOv5 has four versions, namely YOLOv5s, YOLOv5m, YOLOv5l, YOLOv5x. From YOLOv5s to YOLOv5l, the depth and width of the model are increasing, and the number of parameters is also enlarging. Although the accuracy will be improved with the increase in the complexity of the model, it also has a great impact on the detection speed. YOLOv5l and YOLOv5x are excluded due to their large number of parameters that would greatly reduce the detection speed. YOLOv5 is composed of several convolution layers. The convolution of each part has its own depth and width. The depth coefficient of YOLOv5s is 0.33, and the width coefficient is 0.5. The meaning of the depth coefficient is to control the depth of the network by controlling the number of stacking layers. The width coefficient controls the width of the network by controlling the number of convolution output channels. By limiting the depth and width, we can control the number of parameters of the whole network. A feature map attention (FMA) layer, which is inserted between the backbone and neck, is proposed to improve the network structure based on YOLOv5s, as shown in Figure 3. In the FMA layer, the feature map of each channel of the attention feature map (shown as four channels in Figure 3) and the input feature map is weighted to obtain the four groups’ features. Then, 1 × 1 convolution is used to downsample the number of channels for each group’s feature map. Eventually, the four groups’ feature map after channel downsampling is concatenated to obtain a new output feature map. Since the channel downsampling multiple of the feature maps’ attention layer is the same as that of the attention feature map, the input and output feature map have the same size.

### 2.3. Network Training

**Figure 4:**
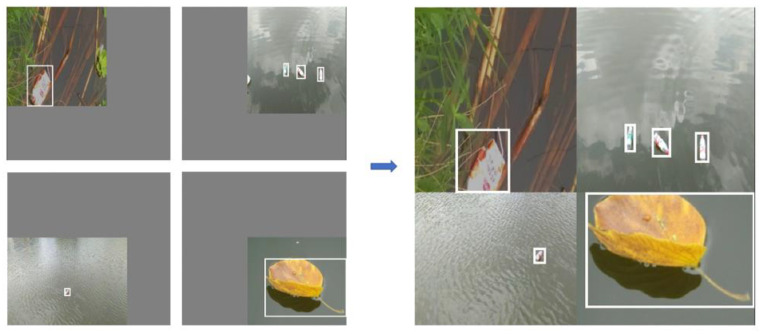
The mosaic data augmentation.

The network training in this paper is completed by the FMA-YOLOv5s algorithm, and the training process is as follows: Step 1, data preprocessing. The mosaic data augmentation method is used, as shown in Figure 4. Step 2, input a batch of image data into the network for the forward propagation to obtain the detection result. Step 3, compare the detection results with the label value and then calculate the loss value. Step 4, backpropagate based on the loss value and update the weight according to the learning rate. Step 5, repeat steps 2, 3 and 4 until the network loss continues to decrease and tends to converge.

**Figure 5:**
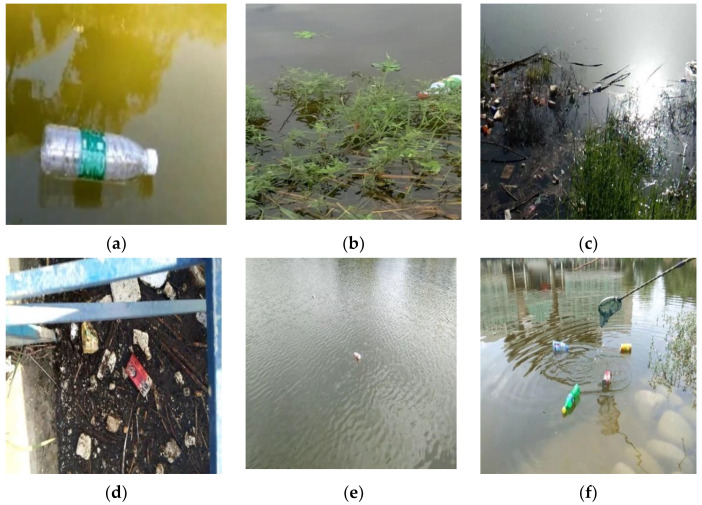
Float debris images with various disturbance: (**a**) ideal detection target; (**b**) blocked target; (**c**) strong reflection; (**d**) complex water surface; (**e**) small target; (**f**) ripples.

In order to realize the real-time detection of the floating debris in the river environment, the data required for modeling are collected in the actual river channel. The specific image data include the following situations, as shown in Figure 5: water grass or other facilities block a part of the monitoring target, strong sunlight reflection near the floating objects, complex water surface of the river, too small target in the image, ripple near the target, etc. The above situations are difficult examples in the monitoring of floating debris, and adding such images can improve the robustness of the model.

The mosaic data enhancement method is used by FMA-YOLOv5s based object detection algorithm to preprocess the video data to solve these problems in this paper. This method refers to the CutMix method. CutMix uses two images for splicing, and the mosaic uses four images, which can enrich the background of the detected object. Mosaic technology can compute the data of four images at a time. The process is as follow: first, randomly select four images; secondly, flip, zoom and color gamut changes of the four pictures, respectively, and place them in four directions; next, combine the images and frames, and re-splice the four images into a new image in the order of upper left, lower left, lower right and upper right, respectively; finally, use this image data for training.

The effect of mosaic data augmentation processing of this algorithm is shown in Figure 4. The advantage is that, firstly, it is equivalent to increasing the number of training images each time, which is conducive to saving the GPU memory of the training equipment and also increases the number of images in each batch; secondly, it is conducive to the training model’s ability to detect small targets. The target object in each image becomes smaller than the whole image by splicing four images into one. This method is helpful to improve the model’s ability to detect smaller targets in the waterway.

### 2.4. Detection Process

**Figure 6:**
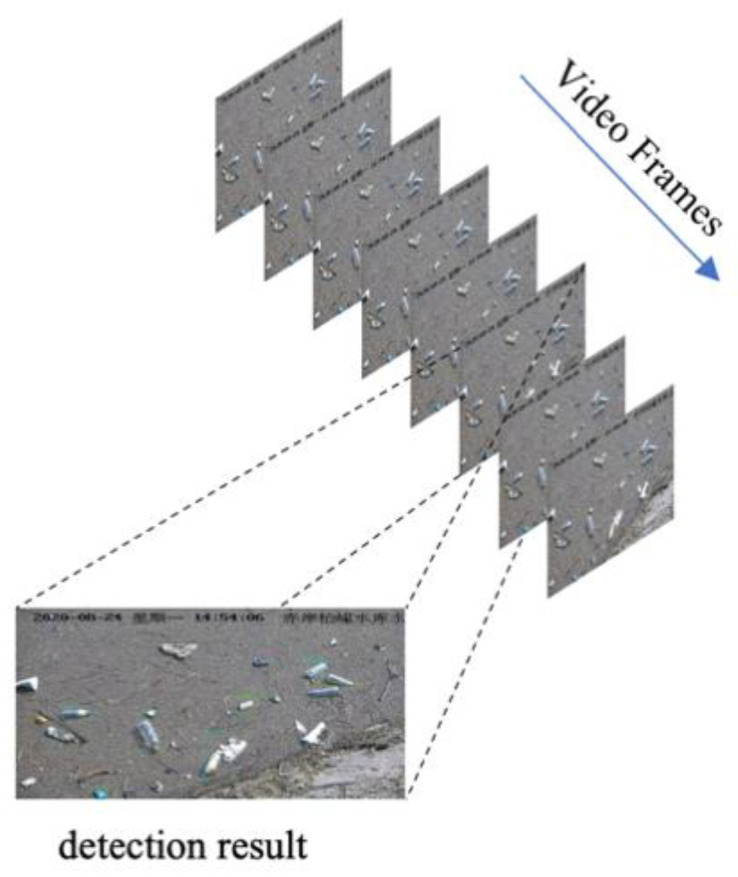
Frame by frame detection.

The flow of the detection process is as follows: Step 1, take the image to be tested as input and extract the features of the image through the backbone. Step 2, extract the feature maps of different depths of the backbone network. Step 3, the extracted multi-scale feature maps are used as the input of the FPN structure for feature fusion. As an improvement, the upsampling of the feature map is a bilinear interpolation method. Step 4, the multi-scale feature maps after FPN fusion are input into the PAN structure for strong feature localization, and the detection results of three feature maps with different scales are obtained. Step 5, after all feature map detection results are processed by nms, the final results will be generated, and detection boxes and categories will be labeled in the original input images. Step 6, extract the next frame of the image to be detected and repeat steps 1 to 5 to complete the video frame by frame detection, as shown in Figure 6.

### 2.5. Loss Function

The loss function is an important index to measure the similarity between the training and the real results. The output of the YOLO algorithm model predicts the center coordinate, width and height of the bounding box. In the algorithm of YOLOv3, the loss function calculates the mean square error (MSE) of the center coordinate, width and height, respectively. Because the YOLOv3 algorithm does not consider the relationship between the two kinds of results, the loss function cannot truly reflect the difference between the predicted and the real value, which affects the performance of the model. YOLOv4 and YOLOv5 algorithms change the loss function of the prediction bounding box to *CIoU* [37] function, which considers the scale information of the coincidence degree, center distance and aspect ratio of the border on the basis of *IoU*, as is shown in the following equation:

(1) $$L_{IoU}=1-IoU\left(Box_{pre},Box_{gt}\right)$$

where $Box_{pre}$ and $Box_{gt}$ are the predicted bounding box and the real bounding box, respectively, and they are the overlapping area.

(2) $$L_{CIoU}=1-IoU\left(Box_{pre},Box_{gt}\right)\;+\;\frac{\rho^{2}\left(Box_{pre\_ctr},Box_{gt\_ctr}\right)}{c^{2}}+\alpha .v$$

(3) $$\alpha =\frac{v}{\left(1-IoU\right)\;+\;v}$$

(4) $$v=\frac{4}{\pi^{2}}{\left(\arctan\frac{w_{gt}}{h_{gt}}-\arctan\frac{w_{pre}}{h_{pre}}\right)}^{2}$$

where α is a positive number, and v is a penalty term to measure the wide and high similarity between the predicted value and real value. $w_{gt}$, $h_{gt}$, $w_{pre}$ and $h_{pre}$ are the width and height of the real and predicted values of the bounding box, respectively. The middle term of the loss function is the penalty term to measure the distance between the center points, where the $\rho \left(\cdot \right)$ is the Euclidean distance, $Box_{pre\_ctr}$ and $Box_{gt\_ctr}$ are the center coordinate of the predicted value and the real value, and c is the diagonal length of the minimum bounding box between the predicted and real bounding boxes. Moreover, YOLOv5 adopts a cross neighborhood grid matching strategy (one Ground Truth can match multiple anchors) in the definition of positive and negative samples so as to get more positive sample anchors and accelerate the convergence of the loss function.

## 3. Experiments and Results

### 3.1. Dataset and Expand Method

**Figure 7:**
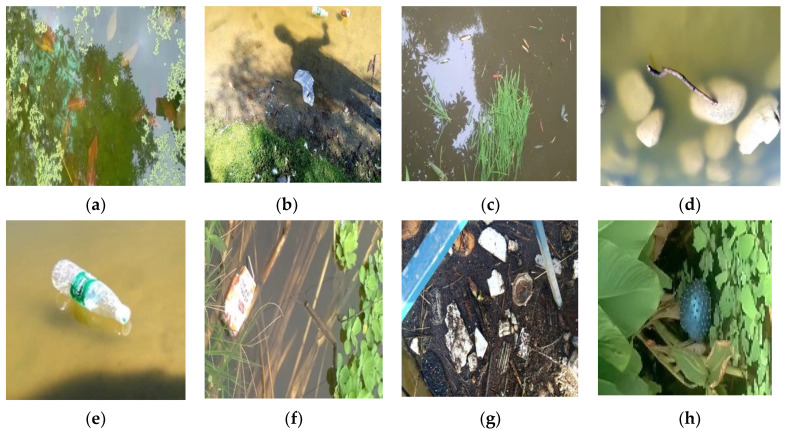
Floating debris category: (**a**) leaf; (**b**) plastic-bags; (**c**) grass; (**d**) branch; (**e**) bottle; (**f**) milk box; (**g**) plastic garbage; (**h**) ball.

Since there is no available relevant large dataset of floating debris in the waterway, the dataset used in this paper is derived from the floating debris images collected by the project. The total number of images is 2400, including 8 categories: leaf, plastic bags, grass, branch, bottle, milk boxes, plastic garbage and a ball, as shown in Figure 7. The above eight categories of floating debris are common floating debris in the river. The image data is labeled with LabelImg labeling tool and is made into VOC format data set, and the label is saved in an xml file. The labeling method of the bounding box is the coordinate values of the upper left corner and the lower right corner. Since the label format required by the YOLO series algorithm is a txt file, normalized by the center coordinates of the bounding box and the width and height, the dataset format needs to be converted.

**Figure 8:**
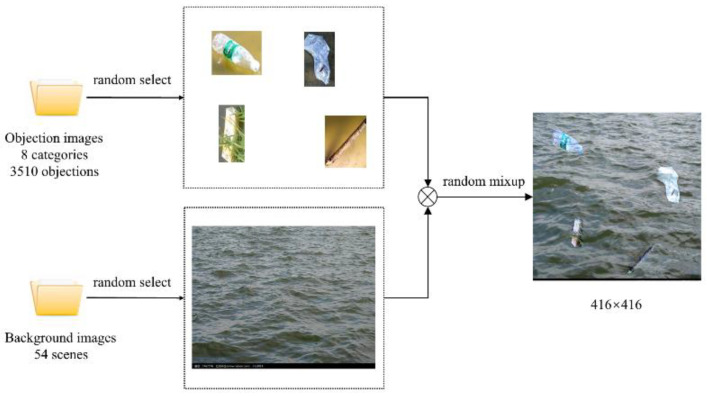
Dataset expanding method.

**Figure 9:**
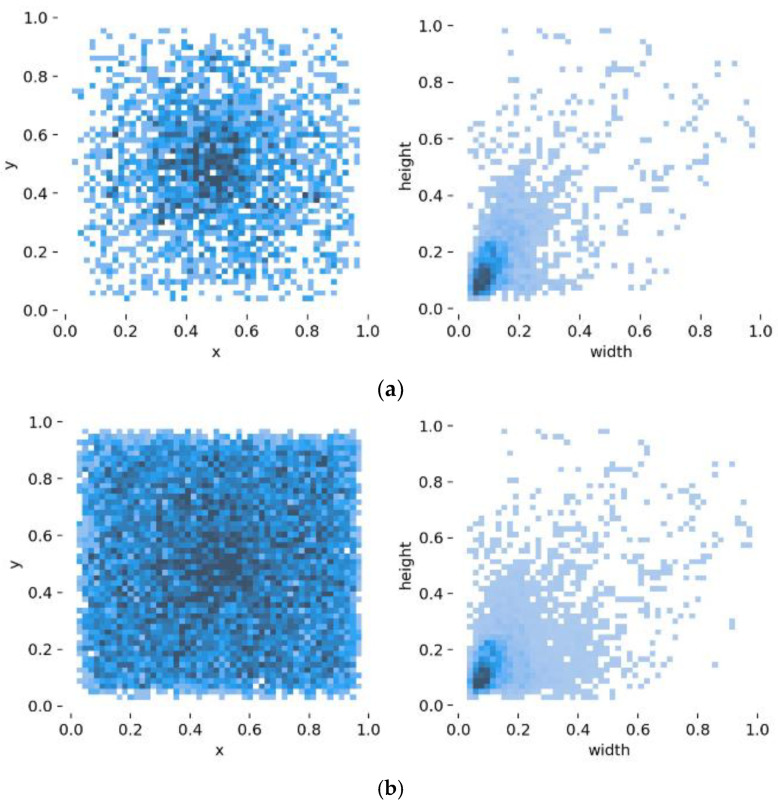
Bounding box distribution. (**a**) Dataset-original; (**b**) Dataset-expanded.

Due to the randomness of the occurrence time of river floating debris, it is difficult to collect a large number of training datasets in the actual environment in a short time. An effective method to expand the training dataset of river floating debris is presented. This approach can alleviate the problem of insufficient training data set to some extent and enhance the detection effect of a small target. The labeled target objects are extracted from the original train dataset, and a total of 2824 target objects are extracted from 1920 images. Then 54 background images of the clean river surface in the actual scene are collected, including clear and turbid water, various water color and different light conditions. Furthermore, a background image is randomly cut into a 416 × 416 image, and the target objects and background image are selected randomly to get a new training image containing various target objects. The image generation process is shown in Figure 8. The Poisson Blending [38] method is applied to merge the target objects and background image, and it is called seamlesClone function in OpenCV. The training dataset is expanded by this approach from 1920 to 4800, and the test dataset is 480. According to the comparison of the center point coordinates and bounding box size of the target object in the dataset, it was found that the overall distribution trend of the target object does not change after expansion, and there are more small objects in the dataset (see Figure 9).

### 3.2. Model Training Results and Analysis

It is necessary to perform scale clustering processing on all labeled borders in the dataset, and the method used is the K-Means clustering algorithm. The flow of the algorithm is as follows: Step 1, randomly select nine of all the labeled Ground Truth sample points as the cluster center (each sample is a four-dimensional vector); Step 2, calculate the distances from all other sample points to these nine centers, respectively, and each sample point belongs to the center point nearest to it; Step 3, in the newly divided clusters, a new cluster center is chosen by means of the average value of four dimensions; Step 4, repeat step 2 and 3 until the new cluster center does not change from the previous cluster center or the change is limited within the specified range.

The distance uses the Euclidean distance equation:

(5) $$dist\left(X,Y\right)\;=\;\sqrt{\overset{4}{\underset{i=1}{\sum }}{\left(x_{i}-y_{i}\right)}^{2}}$$

The clustering results are sorted by area size as (23, 29), (37, 34), (26, 53), (41, 53), (41, 90), (94, 40), (61, 75) and (78, 135). It can be found that the scales of the prior boxes are quite different, and they are assigned to 76 × 76, 38 × 38, 19 × 19 three feature maps as the prior boxes.

The deep learning framework used in this experiment is Pytorch, the training platform is Ubuntu 18.04, the CPU is Intel i9-10900k, and the GPU is a single-card NVIDIA GeForce GTX 2080Ti (11GB). The batch size is set to four, and the epoch was set to 300. Adam is used to optimize the network. The decay method of the learning rate is to multiply the learning rate by the coefficient γ=0.9 after a certain step.

The mean average precision (mAP) is used as the evaluation index of the model performance.

(6) $$P=\frac{TP}{TP+FP}$$

(7) $$R=\frac{TP}{TP+FN}$$

(8) $$\mathrm{mAP}=\frac{1}{N}\overset{N}{\underset{i=1}{\sum }}{\mathrm{AP}}_{i}$$

where, *P* is precision rate, *R* is recall rate, *TP* is True Positives, *FP* is False Positives, *FN* False Negatives. ${\mathrm{AP}}_{i}$ is the area under the *P*(Precision) − *R*(Recall) curve of a certain class i, which is obtained by adjusting the threshold values of some columns to the images drawn with different *P* and *R* values. The mAP can be obtained by adding and averaging AP values under each corresponding category, and it can reflect the overall performance of the model.

### 3.3. Model Training Results and Analysis

In order to verify the effectiveness of this algorithm, other six network structures such as SSD, YOLOv2, YOLOv3, YOLOv4, YOLOv5s and YOLOv5m are selected for comparative experiments. Table 1 shows the performance of these seven models on two train datasets. The table shows that the accuracy of all models is better than the previous models in the expanded training dataset. Moreover, YOLOv4 and YOLOv2 increased significantly by 3.6% and 2.54%, respectively. The performance of YOLOv2 is obviously worse than that of YOLOv3 in the detection of floating debris. The reason is that there are no multi-scale feature maps in YOLOv2, which makes some small targets easy to be ignored. The performance of SSD, YOLOv3 and YOLOv4 with multi-scale feature maps is similar. In terms of detection speed, YOLOv5s performs the best one, FMA-YOLOv5s is close to YOLOv5s, only 1 FPS lower. YOLOv4 performs the worst because it has the largest number of parameters. On the whole, the mAP of the YOLOv2 model is the lowest, which is 71.23%. In a word, when considering both detection speed and accuracy, FMA-YOLOv5s has better performance.

The results of a series of comparative experiments on the YOLOv5s are shown in Table 2. Those experiments consider the influence of three factors of data set expansion, mosaic data augmentation and FMA layer, separately or in various combinations. The AP of each category is shown in detail. In Table 2, the first row is chosen as the baseline. Comparing the second row with baseline, the mAP increased by 0.29%, and the AP value of the plastic bag category increased obviously. The mAP values of the third row are 2.47% and 2.18%, higher than those of the first and second row, respectively, and the AP values of the eight categories are better. The FMA-YOLOv5s, which is trained by mosaic data augmentation on the expanded dataset, has the best comprehensive performance.

**Figure 10:**
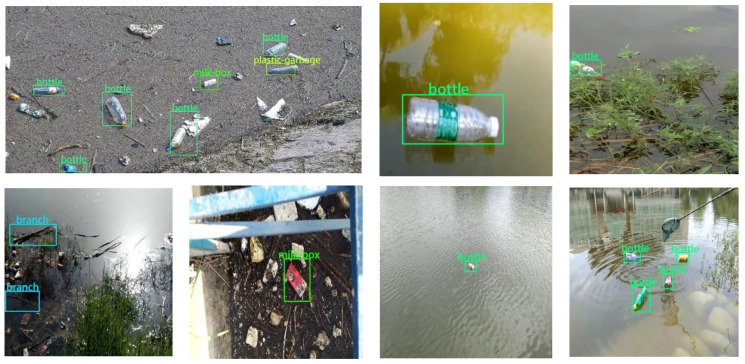
Actual test results.

The real-time water quality data is collected by the camera to detect the application effect of the algorithm in the actual project. As shown in Figure 10, the monitoring algorithm can still correctly detect floating debris when the water body is covered with a lot of floating objects, and some images in the test set are displayed visually.

## 4. Conclusions

The FMA-YOLOv5s network architecture is proposed in this paper. On the basis of YOLOv5s, a feature map attention (FMA) layer is added at the end of the backbone to improve the ability of feature extraction. FMA layer uses a self-attention mechanism to weigh each channel of the upper layer feature map and uses 1 × 1 convolution to control the number of output channels to keep consistent with the input without increasing the computation of the neck part. In the neck part, FPN and PANet are used to enhance the fusion between features. A data expansion method is introduced to expand the training dataset from 1920 to 4800. This approach merges the labeled target objects extracted from the original training dataset and the background images of the clean river surface in the actual scene. The strategy of mosaic data augmentation is applied to raise the mAP value of the model and enhance the detection effect of small targets. The FMA-YOLOv5s model obtains the mAP of 79.41% and 42 FPS on the test dataset, wherein the mAP value exceeds YOLOv5s by 2.18%. This algorithm can meet the requirements of the rapidity and accuracy in real-time monitoring of floating objects in waterways. Due to the strong robustness of the deep learning algorithm for water grass occlusion, water surface reflection and other special cases, it is suitable for most environments. This algorithm successfully applies the machine learning method to the monitoring of floating debris in the waterway of urban and rural rivers, which can greatly enhance the efficiency of automatic monitoring of floating debris and save a lot of manual work. It can be widely used to monitor the floating objects in urban inland rivers and is of great significance to environmental protection. More attention can be paid to improving the detection of blurred and dense objects and support more floating debris categories in the future. Meanwhile, the semi-supervised learning method could be considered to reduce the workload of manual annotation and make the model more robust in the follow-up researches.

## Figures and Tables

**Table 1:** Comparison of the results with the other six models.

Model	Input	mAP(%)		FPS	Params (M)
		Dataset-Original	Dataset-Expanded		
SSD	416 × 416	72.06	73.14	26	105
YOLOv2		68.69	71.23	25	193
YOLOv3		73.95	74.87	24	248
YOLOv4		71.75	75.35	22	244
YOLOv5s		76.12	77.23	43	13.7
YOLOv5m		75.79	78.26	39	40.5
FMA-YOLOv5s		77.83	79.41	42	18.2

**Table 2:** Comparison of the result in detail.

Method	Dataset-Expand	Mosaic	FMA	AP(%)								mAP (%)
				Bottle	Milk-Box	Ball	Plastic-Bag	Plastic-Garbage	Branch	Grass	Leaf	
YOLOv5s	√			92.73	86.82	83.01	81.21	82.79	60.74	62.88	65.23	76.94
	√	√		93.92	83.44	85.52	87.72	87.24	55.69	59.71	65.23	77.23
	√	√	√	93.81	85.74	88.68	85.83	85.97	66.98	62.46	66.23	79.41
